# The Significance of Sonication in the Detection of Peri-Implant Infections

**DOI:** 10.3390/antibiotics15010035

**Published:** 2026-01-01

**Authors:** Christian Prangenberg, Alberto Alfieri Zellner, Jonas Roos, Lisa Fiona Roder, Soufian Ben Amar, Kristian Welle, Frank Sebastian Fröschen, Gunnar Thorben Rembert Hischebeth

**Affiliations:** 1 Department of Orthopedics and Trauma Surgery, University Hospital Bonn, 53127 Bonn, Germany; 2 Institute of Medical Microbiology, Immunology and Parasitology, University Hospital of Bonn, 53127 Bonn, Germany

**Keywords:** sonication, periprosthetic joint infections, pseudarthrosis

## Abstract

**Objective**: The current gold standard for detection of implant-related infections is the intraoperative collection of tissue samples. However, false-negative results frequently occur, particularly in infections caused by biofilm-forming bacteria. As a complementary method, sonication has therefore been established for detecting implant-associated infections, especially in periprosthetic joint infections. In trauma surgery, this technique is still rarely used. The aim of this study is to evaluate the diagnostic significance of sonication after osteosynthesis. **Methods**: A retrospective single-center analysis was conducted on all patients who underwent plate osteosynthesis removal between 1 January 2019, and 1 May 2021, with both sonication and intraoperative tissue sampling performed. Patients with inlying arthroplasties or nail-less osteosynthesis systems were excluded. Pre- and postoperative infection parameters (leukocytes, CRP) were recorded, and preoperative clinical findings were used to classify suspected infection. **Results**: A total of 57 patients (30 men, 27 women; mean age 57.6 years, range 12–91) were included. The mean treatment duration was 20.1 days (range 1–152). Sonication was positive in 33 patients, tissue samples in 28, with 31 cases (54%) showing concordant results. In cases with preoperative suspicion of infection, sonication was positive in 21 of 26 cases (80.7%) and tissue samples in 18 of 26 (69.2%), whereas without suspicion, positivity rates were 38% and 31%, respectively. Sonication and tissue results matched in 14 of 26 cases; in the remainder, results were different or incomplete. Preoperative CRP elevation and the presence of an infection membrane influenced sensitivity: sonication generally detected more bacteria (59–81%) than tissue samples (49–73%), though discrepancies remained. **Conclusions**: Sonication represents a valuable complementary method for detecting implant-related infections. Due to its high sensitivity but limited specificity, it should not be used as a standalone diagnostic method.

## 1. Introduction

Targeted and reliable diagnosis of peri-implant infections represents a particular challenge in orthopedics and trauma surgery. The diagnostic gold standard is the collection and microbiological analysis of, ideally, five representative tissue samples [1,2]. Especially in acute implant-related infections, routine cultures can effectively detect bacteria [3]. On foreign materials such as endoprostheses or osteosyntheses, bacteria can form a polysaccharide-rich, strongly adherent biofilm, from which they are difficult to detach and detect. Furthermore, the transition from free-floating planktonic to sessile bacterial forms complicates antibiotic treatment [3,4,5]. Biofilm formation leads to false-negative results in up to 42% of cases [6,7,8]. To improve bacterial detection in peri-implant infections, sonication of explanted prostheses has been increasingly applied in recent years [3,5,6,7,8]. In sonication, implants are exposed to low-frequency ultrasound waves to dislodge adherent bacteria from the biofilm [3,5,7,8]. The bacteria can then be cultured from the sonicate [3,5,7,8]. Anderson et al. cultured the bacteria obtained from sonication by growing them in blood culture bottles. This procedure complements conventional sonication methods [9]. In the field of periprosthetic infections, sonication is therefore already an established diagnostic adjunct [1,2,3]. Sensitivity can reach 47–78.5% [2,10], and specificity is notably higher compared with tissue samples (99% vs. 80%) [10]. However, multiple studies have demonstrated that sonication results may differ from those of tissue cultures [1,2].

Following fracture treatment with internal fixation, the diagnosis of an implant-related infection is not always rapid or straightforward [6]. These infections often do not present with classic acute signs but rather with chronic pain or the clinical appearance of a pseudarthrosis [6]. Particularly after open fractures, postoperative infection rates of up to 30% have been reported, with sometimes delayed diagnosis and initiation of targeted therapy [7,11]. This also applies to pseudarthroses without clinical suspicion of infection [4].

The present study aims to demonstrate that sonication is a diagnostically suitable method for detecting peri-implant infections, comparable in quality to its established use in periprosthetic infections.

## 2. Results

During the study period, a total of 57 patients who underwent plate removal with microbiological diagnostics (sonication and tissue sampling) were included. Among these, 30 were men (52.6%) and 27 were women (47.4%). The mean age at admission was 57.6 years (range 12–91, SD 19.78). The mean treatment duration was 20.1 days (range 1–152, SD 28.1).

**Figure 1:**
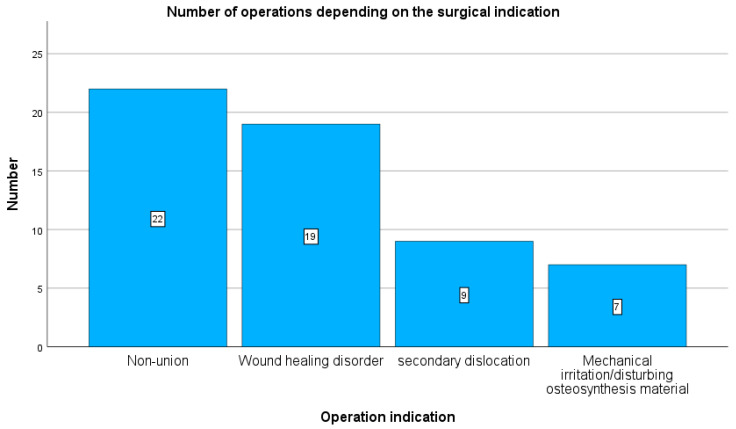
Indication for plate removal. The majority of procedures were performed due to pseudarthrosis and wound-healing disturbances. Additional indications included secondary dislocation and mechanical irritation caused by the implanted osteosynthetic plate.

Plate removal was performed due to pseudarthrosis in 22 cases (38.5%), wound healing disorder in 19 cases (34%), secondary screw dislocation in 9 cases (15.7%), and mechanical irritation in 7 cases (12.5%) (see Figure 1).

**Figure 2:**
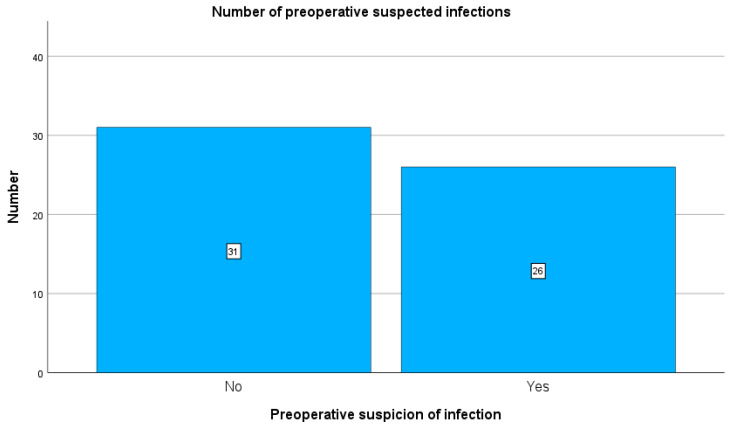
Representation of the two study groups. Of the 57 included patients, 31 had no preoperative suspicion of infection, while 26 showed a preoperative suspicion of infection.

Preoperatively, 26 patients were clinically suspected of having an implant-related infection, whereas 31 patients had no preoperative clinical suspicion of infection (see Figure 2). CRP elevation > 3 mg/L was observed in 37 patients, while 20 patients had CRP < 3 mg/L.

Pathological analysis was additionally performed in 43 patients. No infection membrane was detected in 17 cases, while 26 cases showed an infection membrane.

**Figure 3:**
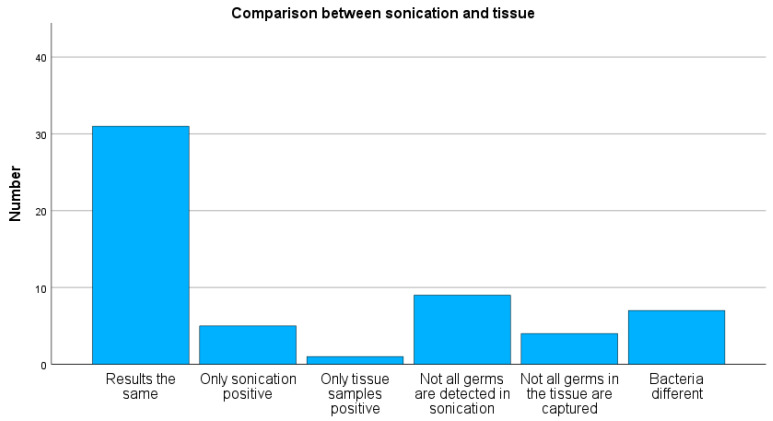
Analysis and comparison of microbiological samples obtained from tissue and sonication. In the majority of cases, concordant results were observed between sonication and tissue samples.

A positive bacterial detection by sonication was observed in 33 cases, while 24 cases were negative. Tissue cultures showed positive bacterial growth in 28 cases and no detection in 29 cases. Concordant results between sonication and tissue cultures were found in 31 of 57 cases (54%) (see Figure 3). In five cases, bacterial growth was detected only in sonication, and in one case only in tissue culture. Sonication revealed not all the same bacteria as tissue cultures in nine cases, whereas tissue cultures detected not all the same bacteria as sonication in four cases. Discordant bacterial growth was observed in seven cases.

**Figure 4:**
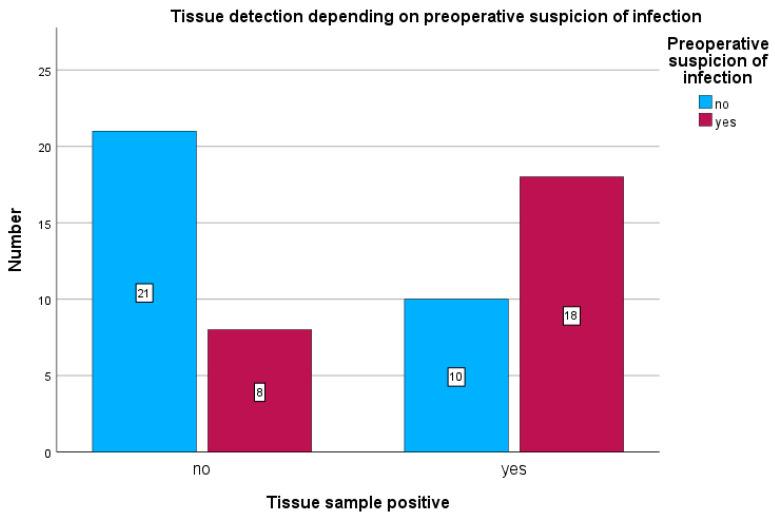
Detection of bacteria in tissue samples in relation to preoperative clinical suspicion of infection. The chart shows the number of patients with positive tissue cultures depending on whether a preoperative suspicion of infection was present (yes) or absent (no). Blue bars represent patients without preoperative infection suspicion, and red bars represent patients with preoperative infection suspicion.

**Figure 5:**
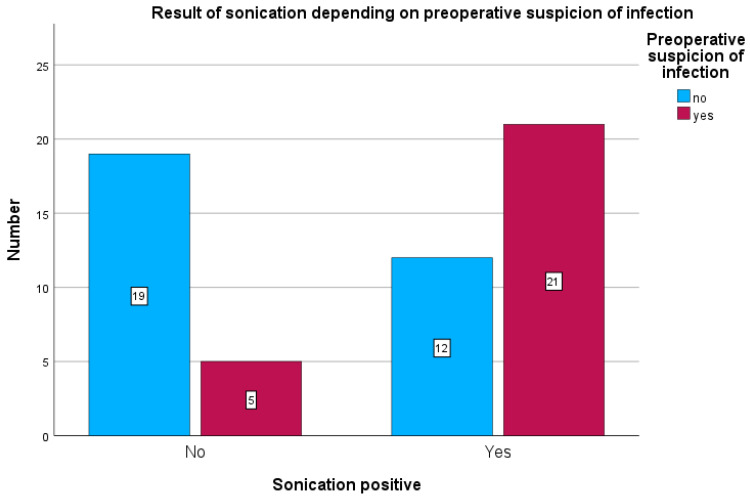
Positive bacterial detection in sonication depends on preoperative suspicion of infection.

Among patients with preoperative clinical suspicion of infection, tissue cultures were positive in 18 of 26 cases (69.2%) (see Figure 4) and sonication was positive in 21 of 26 cases (80.7%) (see Figure 5). Sonication was negative in 5 of 26 cases (19.2%), and tissue cultures were negative in 8 of 26 cases (30.7%).

In patients without preoperative suspicion of infection, sonication was positive in 12 of 31 cases (38%) and tissue cultures in 10 of 31 cases (31%).

**Figure 6:**
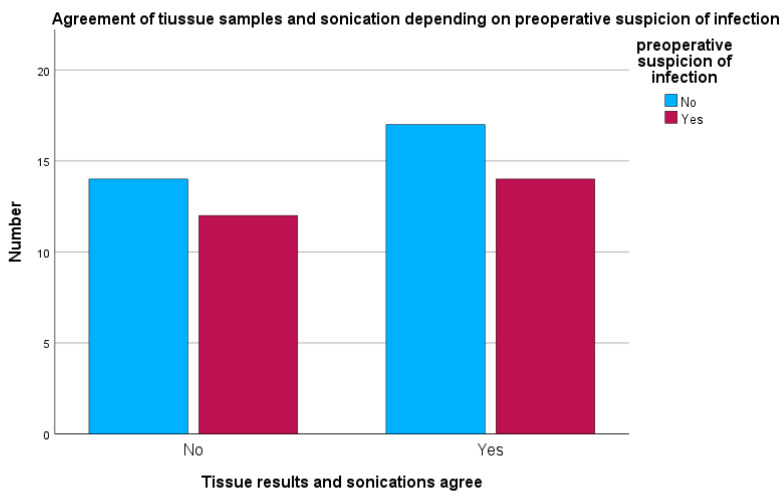
Concordance between sonication and tissue samples depending on preoperative suspicion of infection.

When preoperative suspicion of peri-implant infection was present, sonication and tissue cultures were concordant in 14 of 26 cases (53.8%). In 12 of 26 cases (46.2%), sonication and tissue cultures were discordant, showing different microbiological results (see Figure 6).

**Figure 7:**
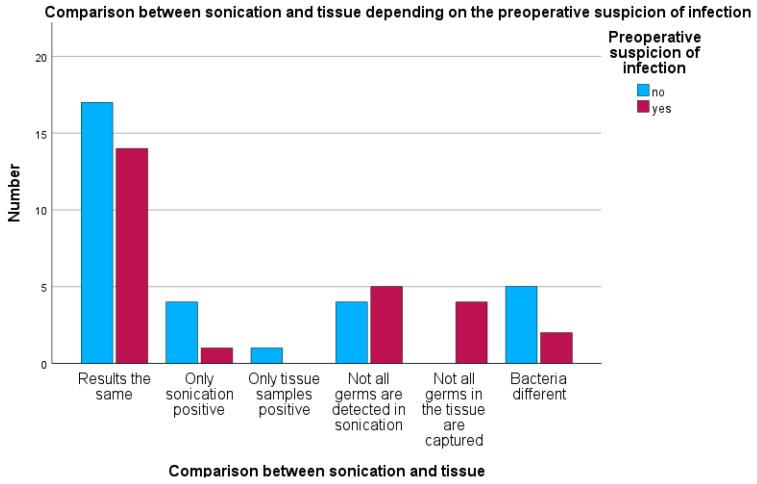
Differences between sonication and tissue samples depend on preoperative suspicion of infection.

In the group with clinical preoperative infection suspicion, two cases each showed positive bacterial detection in both sonication and tissue cultures, although the bacteria detected were different (7.6%). In one case, only sonication detected bacteria despite preoperative suspicion (3.8%). In five cases, tissue cultures detected more microorganisms than sonication (19.2%), while in four cases, sonication detected bacteria not found in tissue cultures (15.3%) (see Figure 7).

Of the 26 cases with preoperative infection suspicion, 14 cases (approximately 54%) showed identical results between sonication and tissue cultures. In only one case (4%) was sonication exclusively positive, while there were no cases with tissue cultures exclusively positive. In five cases (19%), sonication did not detect all bacteria, whereas in four cases (15%), tissue cultures were incomplete. In two further cases (8%), bacteria were detected by both methods but were different species.

In patients with preoperative CRP elevation, sonication was positive in 22 of 37 cases (59.4%), and tissue cultures were positive in 18 of 37 cases (48.6%). Sonication was negative in 15 of 37 cases (40.5%), and tissue cultures were negative in 19 of 37 cases (51.3%).

In patients without preoperative CRP elevation, sonication was positive in 11 of 20 cases (55%), and tissue cultures were positive in 10 of 20 cases (50%).

When an infection membrane was detected intraoperatively, sonication was positive in 21 of 26 cases (80.7%), and tissue cultures were positive in 19 of 26 cases (73.1%). Sonication was negative in 5 of 26 cases (19.2%), and tissue cultures were negative in 7 of 26 cases (26.9%).

In cases without an infection membrane, sonication was positive in 6 of 17 cases (35.3%), and tissue cultures were positive in 3 of 17 cases (17.6%).

## 3. Discussion

Sonication has been an established tool with very high sensitivity for the detection of periprosthetic joint infections for several years [2,8,12]. Preoperative administration of antibiotics does not affect the results [5]. However, sonication is not yet routinely used for the identification of peri-implant infections [13,14,15].

Studies report that sonication achieves a sensitivity of 47–78.5% in periprosthetic joint infections [2,10,16], which aligns with our results. For this reason, sonication serves as a useful adjunct for detecting periprosthetic infections but cannot be used as a sole diagnostic method. Consequently, tissue sampling remains clinically valuable and continues to represent the gold standard for the diagnosis of such infections [6,17,18,19].

In the present study, microbiological data from 57 patients are retrospectively analyzed from both sonication of an osteosynthesis plate and corresponding tissue samples to evaluate the suitability of sonication for detecting peri-implant infections. The study was conducted using a retrospective design. Patients were divided into two groups: in cases with classical signs of inflammation, such as redness, swelling, warmth, or wound healing disorders, preoperative infection suspicion was present. The comparison group included patients who underwent osteosynthesis removal due to mechanical irritation, without classical inflammatory signs. Additionally, preoperative infection markers and intraoperatively obtained pathological samples were considered. Accordingly, two groups were defined. Overall, results from sonication and tissue samples matched in only 31 of 57 cases. Ponraj et al. also reported differing bacterial yields between sonication and tissue samples [20].

Thus, in just over 50% of cases, sonication and tissue samples showed concordant results.

The analysis demonstrates that sonication detects more infections in cases with clinical suspicion, with a sensitivity of 80.8%, compared to 69.2% for tissue samples. However, sonication has a lower specificity of 61.3%, while tissue samples show slightly higher specificity at 67.7%. Overall, this indicates that sonication is more sensitive for detecting infections but produces more false-positive results. Tissue samples, on the other hand, may miss more infections but provide slightly higher accuracy in correctly identifying non-infected cases.

Furthermore, the data show that sonication has a sensitivity of 59.4% (22/37) in patients with elevated preoperative CRP, while tissue samples reach 48.6% (18/37). In cases without preoperative CRP elevation, sonication demonstrates a specificity of 45.0% (9/20), whereas tissue samples achieve 50.0% (10/20). Therefore, sonication is somewhat more sensitive in detecting infections with CRP elevation but has lower specificity compared to tissue samples. Tissue samples are comparatively more specific but may miss more infections.

In the presence of an infection membrane, sonication shows a sensitivity of 80.7% (21/26), while tissue samples reach 73.1% (19/26). In cases without an infection membrane, sonication demonstrates a specificity of 64.7% (11/17), whereas tissue samples show a significantly higher specificity of 82.4% (14/17).

In summary, sonication is more sensitive in the presence of an infection membrane and therefore detects infections more reliably. However, its specificity is lower, resulting in more frequent false-positive results. Tissue samples, in contrast, have slightly lower sensitivity but considerably higher specificity, making them more reliable for confirming the absence of infection.

Overall, the analysis indicates that sonication has higher sensitivity and detects infections more reliably than tissue samples, whereas tissue samples achieve slightly higher specificity and are better at confirming the absence of infection. In cases of clinical infection suspicion and the presence of an infection membrane, sonication shows significantly higher sensitivity but lower specificity, resulting in more false-positive results. Tissue samples may miss more infections but offer greater accuracy in ruling out infection. The same pattern is observed with preoperative CRP elevation: sonication is slightly more sensitive but less specific than tissue samples. Both methods complement each other diagnostically, with sonication reducing the risk of missed infections and tissue samples providing more reliable confirmation of infection-free status [10,12,21,22].

In 38% of cases without clinical suspicion of infection, positive findings were observed. Predominantly, Staphylococcus epidermidis (skin flora) was detected in both sonication and tissue samples, suggesting the possibility of contamination.

It is critical to note that in 15% of cases, sonication detected a polymicrobacterial result as the findings in tissue samples. In one additional case, only sonication was positive while the tissue sample was sterile. Thus, false-positive results from sonication cannot be excluded in up to 20% of cases.

Sonication can therefore be used as a complementary method for detecting peri-implant infections. Due to its moderate sensitivity but high specificity, however, it should not be used as the sole diagnostic tool [10,23].

Additionally, due to its susceptibility to contamination, results should be interpreted with caution.

## 4. Materials and Methods

This study was conducted as a single-center retrospective cohort analysis evaluating all removals of plate osteosyntheses and subsequent sonication. The observation period was set at three years (from 1 January 2019 to 31 December 2021). The study was performed at a university hospital certified as a supraregional trauma center. The study protocol was reviewed and approved by the responsible ethics committee (Number 406-17).

Since 2017, the examination of all explanted osteosyntheses and endoprostheses has been the established diagnostic standard at the hospital where the study was conducted.

Patients were excluded if implants other than plate osteosyntheses were removed (e.g., intramedullary nails, tension band systems, or endoprostheses). An additional exclusion criterion was patient age < 18 years.

Data were extracted from the hospital’s internal digital database using a procedural search with OPS code 5-787.3. Collected variables included anamnesis data such as age at the time of explantation, sex, surgical indication, and pre-existing comorbidities.

Inclusion criteria comprised the removal of an in situ plate osteosynthesis followed by sonication and simultaneous intraoperative tissue sampling with more than one sample. The specificity and sensitivity of sonication and tissue cultures were investigated. For the detection of infection, clinical wound conditions, preoperative inflammatory markers, and pathological findings were included. Clinical signs of inflammation, such as redness, swelling, local hyperthermia, and exudative wounds, were considered indicative of infection. Additionally, visually exposed osteosynthetic material was regarded as confirmatory for infection.

The tissue samples, which were collected intraoperatively, were shredded, homogenized, and then cultured on Columbia agar with 5% sheep blood, MacConkey agar, chocolate agar, and Sabouraud agar (Becton & Dickinson, Bergen County, NJ, USA). This was also performed with 0.5 mL of sonication fluid. Additionally, 1 mL of the sample was transferred into thioglycolate broth (Becton & Dickinson, Bergen County, NJ, USA). Schaedler and kanamycin/vancomycin agar plates (Becton & Dickinson, Bergen County, NJ, USA) were used for anaerobic cultures. These were streaked with shredded and homogenized intraoperative tissue samples. For the evaluation of the sonication fluid, 0.5 mL of sonication fluid was streaked on culture plates. The incubation conditions of the cultures were 5% CO_2_ at 35 °C for a minimum of 14 days. In addition to the analysis of culture growth, the sonication fluid was added to PEDS medium blood culture flasks (Becton & Dickinson, Bergen County, NJ, USA) and incubated for 14 days in a Bactec FX blood culture system (Becton & Dickinson, Bergen County, NJ, USA). Similarly, the preoperative joint aspiration fluid was inoculated into PEDS medium blood culture flasks (Becton & Dickinson, Bergen County, NJ, USA) and incubated for 14 days in a Bactec FX blood culture system (Becton & Dickinson, Bergen County, NJ, USA). The microbiological procedures were performed according to the methods described by Fröschen et al. (2022) [24].

The aerobic and anaerobic microbiological results of tissue samples and sonication after implant removal were recorded and analyzed. Tissue culture results were subsequently compared with sonication results. Preoperative and postoperative infection parameters (leukocytes, CRP) were collected. Furthermore, pathological analysis was performed to evaluate the presence of an infection membrane.

### 4.1. Statistical Analysis

Statistical analysis was performed using IBM SPSS Statistics 25 (Ehningen, Baden-Württemberg, Germany). Means, standard deviations, 95% confidence intervals (CI: 95%), medians, and the second and third quartiles were calculated. For comparison of parametric values before and after surgery, a paired *t*-test was used for normally distributed data. The Welch test was used for comparisons between two groups of parametric values. Cohen’s effect size was calculated and classified as follows: d = 0.2 small effect, d = 0.5 medium effect, and d = 0.8 large effect. Correlations were determined using Pearson’s method, including effect size according to Evans. The correlation coefficient r was graded as follows: <0.2 = poor, 0.2–0.4 = weak, 0.4–0.6 = moderate, 0.6–0.8 = strong, and >0.8 = optimal. The significance level was set at *p* < 0.05.

### 4.2. Limitations

This study has several limitations. Its retrospective single-center design may introduce selection and information bias and limit generalizability. The lack of a universally accepted gold standard for diagnosing implant-associated infection required reliance on a composite definition based on clinical signs, laboratory parameters, and pathology, which may introduce variability. And finally, the sample size and observational period may limit statistical power to detect rare events or subtle differences between diagnostic methods.

## Data Availability

The original contributions presented in the study are included in the article, further inquiries can be directed to the corresponding authors.
